# Effect of Indocyanine Green Fluorescence Angiography on Anastomotic Leakage in Patients Undergoing Colorectal Surgery: A Meta-Analysis of Randomized Controlled Trials and Propensity-Score-Matched Studies

**DOI:** 10.3389/fsurg.2022.815753

**Published:** 2022-03-15

**Authors:** Gang Tang, Donglin Du, Jie Tao, Zhengqiang Wei

**Affiliations:** ^1^Department of Gastrointestinal Surgery, The First Affiliated Hospital of Chongqing Medical University, Chongqing, China; ^2^Department of Hepatobiliary Surgery, Chongqing Medical University, Chongqing, China

**Keywords:** indocyanine green fluorescence angiography, anastomotic leakage, colorectal surgery, meta-analysis, randomized controlled trial

## Abstract

**Background:**

Meta-analyses have demonstrated that indocyanine green (ICG) can effectively prevent anastomotic leakage (AL) after colorectal surgery. However, recent evidence from large randomized controlled trial (RCT) has suggested that ICG fluorescence angiography does not reduce the incidence of AL in colorectal surgery. This study was conducted to evaluate the value of ICG for the prevention of AL following colorectal surgery.

**Methods:**

Up to September 16, 2021, PubMed, Embase, China National Knowledge Infrastructure, Web of Science, Scopus, Cochrane Library, and VIP databases were searched for RCTs and propensity-score matched (PSM) studies evaluating the use of ICG for prevention of AL after colorectal surgery. Mean differences (MDs) or odds ratios (ORs) and 95% confidence intervals (CI) were calculated.

**Results:**

Twenty studies (5 RCTs and 15 PSM studies) with a total of 5,125 patients were included. ICG did not reduce the reoperation rate (OR, 0.71; 95% CI, 0.38, 1.30), conversion rates (OR, 1.34; 95% CI, 0.65, 2.78), or mortality (OR, 0.50; 95% CI, 0.13, 1.85), but ICG did reduce the incidence of AL (OR, 0.46; 95% CI, 0.36, 0.59) and symptomatic AL (OR, 0.48; 95% CI, 0.33, 0.71), and reduced the length of hospital stay (MD,−1.21; 95% CI,−2.06,−0.35) and intraoperative blood loss (MD,−9.13; 95% CI,−17.52,−0.74). In addition, ICG use did not increase the incidence of total postoperative complications (OR, 0.93; 95% CI, 0.64, 1.35), postoperative ileus (OR, 1.26; 95% CI, 0.53, 2.97), wound infection (OR, 0.76; 95% CI, 0.44, 1.32), urinary tract infection (OR, 0.87; 95% CI, 0.30, 2.59), pulmonary infection (OR, 0.23; 95% CI, 0.04, 1.45), urinary retention (OR, 1.08; 95% CI, 0.23, 5.04), anastomotic bleeding (OR, 1.53; 95% CI, 0.27, 8.60), anastomotic stricture (OR, 0.74; 95% CI, 0.24, 2.29), or operative time (MD,−9.64; 95% CI,−20.28, 1.01).

**Conclusions:**

ICG can effectively reduce the incidence of AL, without prolonging the operation time or increasing postoperative complications in colorectal surgery.

**Systematic Review Registration:**

www.crd.york.ac.uk/prospero/#recordDetails, identifier: CRD42021279064.

## Introduction

Anastomotic leakage (AL) is one of the most destructive complications of colorectal surgery, which is associated with increased length of hospital stay, hospitalization costs, postoperative morbidity and mortality (1, 2). More worryingly, studies have shown that AL can also harm patient's long-term outcomes (3, 4). The incidence of AL after colorectal surgery is as high as 3–20%, especially in rectal surgery (5, 6). The risk factors for AL include male, age, preoperative chemotherapy and radiotherapy, high ASA score, advanced tumor, malnutrition, smoking, alcoholism, obesity, complications, intraoperative sepsis, immunosuppression, blood loss, prolonged operation time, perioperative blood transfusion, diverticutis and inadequate anastomotic blood supply (6, 7). Adequate blood perfusion is the key to good anastomotic healing (1). Therefore, detection of intestinal segments with poor blood supply during surgery can effectively reduce the incidence of AL. Traditionally, surgeons have assessed the blood supply of the anastomotic site primarily by the color of the intestinal mucosa, marginal bleeding, and palpable arterial pulses in the mesentery (8). However, this assessment strategy is susceptible to the clinician's experience and has low accuracy (9). Therefore, it is urgent to find reliable strategies to evaluate anastomotic perfusion.

Indocyanine green (ICG) is a water-soluble tricarbine compound that rapidly binds to plasma proteins when administered intravenously. ICG can absorb near-infrared light, and fluorescence angiography of ICG enables real-time evaluation of blood perfusion during surgery (10, 11). ICG has been widely used in various surgical procedures (12–14). Several cohort studies have suggested that ICG fluorescein angiography may be a potential strategy for preventing AL after colorectal surgery (15–19). However, baseline data from most cohort studies (15–19) do not match, which has stimulated the interest of investigators in conducting high-quality randomized controlled trials (RCTs) to investigate the effect of ICG on AL prevention. Two large and highly anticipated RCTs (20, 21) published recently have shown that ICG fluorescein angiography does not reduce the incidence of postoperative AL, nor does it reduce postoperative complications or mortality. Existing meta-analyses include either low-quality evidence or a limited number of RCTs, so the results of these meta-analyses (4, 5, 8, 22, 23) are not convincing. Propensity-score matched (PSM) study was able to eliminate baseline differences between the experimental and control groups, there is plenty of evidence that PSM studies are almost equivalent to RCTs in evaluating the efficacy of interventions (24).

In order to resolve the current conflicting findings and overcome the lack of high-quality evidence, we conducted a comprehensive literature search and analyzed data from RCTs and PSM studies to clarify the prophylactic effect of ICG on postoperative anastomotic leakage in colorectal surgery.

## Methods

### Search Strategy

Our meta-analysis was conducted based on the Preferred Reporting Items for Systematic Reviews and Meta-Analyses (PRISMA) statement (25). We successfully registered this study protocol on PROSPERO (registration no. CRD42021279064). The Embase, China National Knowledge Infrastructure, Web of Science, Scopus, PubMed, Cochrane Library, and VIP databases were searched to identify RCTs and PSM studies evaluating the effect of ICG in colorectal surgery from inception to September 16, 2021. There are no language restrictions on retrieval. The search terms were: (stomal leak OR anastomotic leakage) AND (indocyanine green OR ICG). To identify potential relevant trials, the reference lists of all included articles were reviewed.

### Study Selection

Literatures were screened by two independent authors according to the following inclusion criteria: (1) patients undergoing colorectal surgery; (2) intervention with ICG fluorescence angiography; (3) compare with surgeon's judgement visually; (4) the outcomes included any of the following: AL rate, symptomatic anastomotic leakage (SAL) rate, postoperative complications, conversion rates, length of postoperative hospital stay, reoperation rate, blood loss mortality and operative time. (5) the study design was RCT or PSM. Meeting abstract, letters, reviews, Studies involving non-human subjects, and case reports were excluded.

### Data Extraction

The following data were extracted: first author, year, type of study, sample, age, gender, primary disease, type of surgery and outcomes. AL is defined as the communication between the intestinal lumen and the outside due to the defect of the integrity of the intestinal wall at the anastomosis (23). AL can be classified into three different grades: grade A, grade B and grade C. Grade A AL, also known as asymptomatic AL, referred to leakage detected only by imaging examination without clinical manifestations or abnormal laboratory examination. Grade B AL was defined as leakage that requires active intervention but does not require reoperation. Grade C AL was defined as leakage requiring reoperation. Grade B and C AL were referred to as SAL (26). If some necessary information could not be extracted from the article, we would contact the corresponding author to try to obtain the missing data.

### Quality Assessment

The Cochrane Collaboration tool for risk of bias was used to assess the risk of bias in RCTs, including the following domains: (a) sequence generation; (b) allocation concealment; (c) blinding of participants and personnel; (d) blinding of outcome assessment; (e) incomplete outcome data; (f) selective outcome reporting; (g) other potential sources of bias. We used the Newcastle-Ottawa score (NOS) to assess the risk of bias in PSM. Three methodological aspects (selection of participants, groups comparability, and outcome) were assessed using a 9-point scale. During the process of literature retrieval, screening, information extraction and quality assessment, any differences between the two authors (Tang and Du) were discussed and resolved with the third author (Tao).

### Statistical Analysis

For dichotomous data, the odds ratio (OR) and 95% confidence intervals (CIs) was calculated. The mean difference (MD) associated 95% confidence intervals (CI) was calculated for continuous outcome data (27). Heterogeneity was assessed using the chi-square test and I^2^. When *I*^2^ > 50%, heterogeneity was considered significant (28). We selected the random-effects model and carried out all statistical analyses taking into account heterogeneity within and between studies. Subgroup analysis was based on type of surgery (low anterior resection only) and type of study design (RCT only). To evaluate the impact of each study on the pooled effect size, sensitivity analysis was conducted using 1-study excluded approach. Analyses were conducted using Review Manager (RevMan) Version 5.3 (The Nordic Cochrane Centre, The Cochrane Collaboration 2014; Copenhagen, Denmark). Funnel plots was performed to evaluate publication bias. *P* < 0.05 was considered statistically significant.

## Results

### Selected Studies

**Figure 1:**
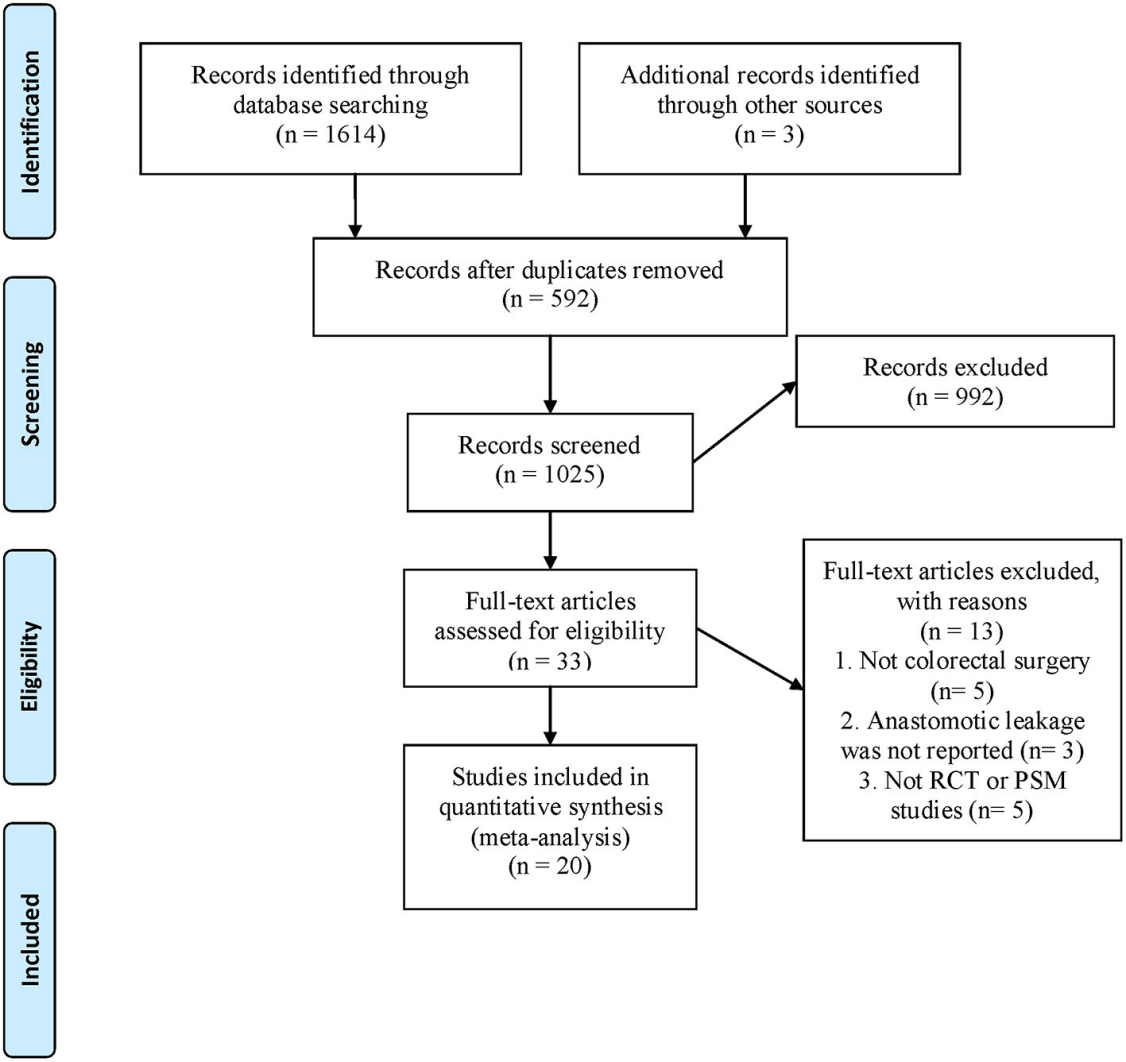
Flow chart of literature search and screening.

A total of 1,617 relevant studies were identified by a preliminary search. After excluding 592 duplicate records, 1,025 articles were eliminated by reading titles and abstracts. Full-text evaluation was conducted in the remaining 33 studies, and finally, 20 studies (20, 21, 26, 29–45) that met the inclusion criteria were included (Figure 1).

### Study Characteristics

Twenty studies (20, 21, 26, 29–45), involving 5,125 participants from 9 countries (United States, Japan, Switzerland, Russia, Italy, France, China, Austria, and Germany), were included in our meta-analysis. Fifteen of the eligible studies (26, 29–32, 34–39, 41, 42, 44, 45) were PSM, while five were RCTs (20, 21, 33, 40, 43). The sample size varied from 54 to 740 subjects. Seven studies (21, 31, 32, 34, 37, 38, 45) performed low anterior resection and the remaining thirteen (20, 26, 29, 30, 33, 35, 36, 38–44) performed colorectal surgery. Follow-up ranged from 30 to 90 days. Most of the studies (21, 29, 31–36, 38, 39, 41–45) included patients only confined to malignant colorectal disease, whereas, five studies (20, 26, 30, 37, 40) included patients with both malignant and benign colorectal disease. Details of the 20 eligible studies (20, 21, 26, 29–45) are summarized in Table 1.

**Table 1:** Characteristics of 20 eligible studies.

**Reference**	**Country**	**Study design**	**Sample**	**Age**	**Gender** **(M/ F)**	**Primary disease**	**Operation method**	**Fluorescence imaging system**	**ICG dose**	**Outcomes**
Kudszus et al. (29)	Germany	PSM	I: 201 C: 201	I: 68 C: 69	I: 85/116 C: 85/116	Colorectal cancer	Colorectal resection	IC-View®, Pulsion Medical Systems AG, Munich, Germany	0.2–0.5 mg/kg	AL rate
Kin et al. (30)	USA	PSM	I: 173 C: 173	I: 58 C: 58	I: 93/80 C: 93/80	Malignant or benign disease	Colectomy or proctectomy	SPY Imaging System (Novadaq Technologies Inc, Bonita Springs, FL)	3ml	AL rate; Reoperation
Boni et al. (31)	Austria	PSM	I: 42 C: 38	I: 69 C: 67	I: 28/14 C: 22/16	Rectal cancer	Laparoscopic LAR	The Karl Storz image1 fluorescence system (Karl Storz, Tuttlingen, Germany)	0.2 mg/kg	AL rate; Reoperation; Postoperative morbidity; Mortality; Operative time; Postoperative hospital stay; No side effects or allergic reaction related to the injection of ICG.
Mizrahi et al. (32)	USA	PSM	I: 30 C: 30	I: 58 C: 58	I: 16/14 C: 18/12	Rectal cancer	Laparoscopic LAR	The PINPOINT™ Endoscopic Fluorescence Imaging System (Novadaq, Toronto, Ontario, Canada)	0.1–0.3 mg/kg	AL rate; Postoperative morbidity; Mortality; Operative time; Conversion rates; No side effects or allergic reaction related to the injection of ICG
Pen et al. (33)	China	RCT	I: 63 C: 82	I: 61 C: 62	I: 36/27 C: 40/42	Colorectal cancer	Colorectal resection	Fluorescent laparoscopic system (Japan, Olympus Corporation)	NA	AL rate; Mortality; No side effects or allergic reaction related to the injection of ICG
Wada et al. (34)	Japan	PSM	I: 34 C: 34	I: 68 C: 67	I: 20/14 C: 24/10	Rectal cancer	Laparoscopic LAR	NIR camera system (PDE-neo System; Hamamatsu Photonics K.K., Hamamatsu, Japan)	5 mg	AL rate; Postoperative morbidity; Mortality; No adverse events related to ICG were observe.
Ishii et al. (35)	Japan	PSM	I: 87 C: 87	I: 64 C: 65	I: 49/38 C: 50/37	Colorectal cancer	Laparoscopic colorectal resection	NA	5 mg	AL rate; No adverse events related to ICG were observe.
Kojima et al. (36)	Japan	PSM	I: 27 C: 27	I: 72 C: 70	I: 15/12 C: 14/13	Colorectal cancer	Laparoscopic left-sided colorectal resection	The LSCI instrument (moorFLPI-2; Moor Instruments, Axminster, UK)	NA	AL rate; Postoperative morbidity; Mortality; Conversion rates; Postoperative hospital stay
Spinelli et al. (37)	Switzerland	PSM	I: 32 C: 32	I: 39 C: 46	I: 21/11 C: 22/10	Malignant or benign disease	LAR	PINPOINT endoscopic fluorescence imaging system (Stryker, Kalamazoo, Michigan, USA),	0.1–0.2 mg/kg	AL rate; Postoperative morbidity; Conversion rates; Operative time; Postoperative hospital stay; Reoperation
Watanabe et al. (45)	Japan	PSM	I: 211 C: 211	I: 66 C: 66	I: 128/83 C: 131/80	Rectal cancer	Laparoscopic LAR	Karl Storz (D-Light P; Tuttlingen, Germany) and the Stryker Corporation (1588 AIM Platform; Michigan, USA)	0.25 mg/kg	AL rate; Postoperative morbidity; Mortality; Operative time; Postoperative hospital stay; Reoperation; Blood loss
Losurdoet al. (39)	Italy	PSM	I: 75 C: 75	I: 71 C: 68	I: 41/34 C: 49/26	Rectal and left colon cancer	Rectal and left colon cancer surgery	A full HD camera system (Karl Storz Image 1-Professional Image Enhancement System-SPIES™, Karl Storz, Germany)	0.2 mg/kg	AL rate; Operative time
Alekseev et al. (40)	Russia	RCT	I: 187 C: 190	I: 63 C: 63	I: 92/95 C: 92/98	Malignant or benign sigmoid or rectal neoplasms	Sigmoid and rectal resection	Laparoscopic system (KARL STORZ GmbH &Co. KG, Tuttlingen, Germany) with light source (D-LIGHT P SCB, KARL STORZ)	0.2 mg/kg	AL rate; Postoperative morbidity; Mortality; Operative time; Postoperative hospital stay; Reoperation; Blood loss
De Nardi et al. (20)	Italy	RCT	I: 118 C: 122	I: 66 C: 65	I: 60/28 C: 66/56	Malignant or benign disease	Laparoscopic left-sided colon and rectal resection	Camera equipped with a xenon light source providing both NIR wavelength and standard light was employed (KARL STORZ GmbH & Co. KG, Tuttlin gen, Germany)	0.3 mg/kg	AL rate; Postoperative morbidity; Mortality; Reoperation; Operative time; Postoperative hospital stay; No adverse events related to ICG were observe
Foo et al. (26)	China	PSM	I: 253 C: 253	I: 67 C: 67	I: 166/87 C: 163/90	Malignant or benign disease	Left-sided colorectal resections	The SPY Elite System (Stryker, USA), Pinpoint System (Stryker, USA)	5–7.5 mg	AL rate; Operative time; Blood loss
Hasegawa et al. (38)	Japan	PSM	I: 141 C: 279	I: 63 C: 63	I: 99/42 C: 203/76	Rectal cancer	Laparoscopic LAR	The IMAGE1 S™ system (Karl Storz SE & Co. KG, Tuttlingen, Germany), 1588 Advanced Imaging Modalities (AIM) Platform and SPY Fluorescence technology (Stryker, Kalamazoo, MI, USA), or HyperEye Medical System Handy (Mizuho Medical Co. Ltd., Tokyo, Japan)	5 mg	AL rate; Operative time; Blood loss; Mortality
Wojcik et al. (41)	France	PSM	I: 42 C: 42	I: 67 C: 69	I: 29/13 C: 29/13	Left-sided colonic or rectal cancer	Left colectomy or anterior resection	NIR light images (FLUOBEAM; Fluoptics, Grenoble, France) or on fusion images merging NIR and standard white light images (PINPOINT; Stryker, Kalamazoo, Michigan, USA)	0.1 mg/kg	AL rate; Postoperative morbidity; Mortality; Operative time; Postoperative hospital stay; Conversion rates
Jafari et al. (21)	USA	RCT	I: 178 C: 169	I: 57 C: 57	I: 104/74 C: 99/70	Rectal cancer	LAR	PINPOINT and/or SPY Elite near infrared range fluorescence imaging (Stryker, Kalamazoo, MI)	7.5 mg	AL rate; Postoperative morbidity; Mortality; Conversion rates
Watanabe et al. (42)	Japan	PSM	I: 370 C: 370	I: 72 C: 72	I: 187/183 C: 187/183	Colon Cancer	Colon cancer surgery	The Stryker Corporation (1588 AIM Platform; MI, USA), Olympus Medical Systems Corporation (VISERA ELITE II, Tokyo, Japan) and Karl Storz (D-Light P; Tuttlingen, Germany).	0.25 mg/kg	AL rate; Postoperative morbidity; Mortality; Operative time; Reoperation; Postoperative hospital stay; Blood loss
Guocong et al. (43)	China	RCT	I: 130 C: 130	I: 68 C: 67	I: 67/63 C: 71/59	Colorectal cancer	Laparoscopic colorectal resection	Fluoroscopy (optomedic-2100)	NA	AL rate; Mortality; Operative time; Postoperative hospital stay; Blood loss
Yanagita et al. (44)	Japan	PSM	I: 93 C: 93	I: N C: N	I: N C: N	Left-sided colon or rectal cancer	Left-sided colon or rectal cancer surgery	near-infrared excitation light (we used mainly Hyper Eye Medical Systems: Mizuho Medical Co., Ltd, Nagoya, Japan and/or IMAGE 1 SPIES™, KARL STORZ SE & Co. KG, Tuttlingen, Germany)	0.1 mg/kg	AL rate; Operative time; Blood loss; Conversion rates

*AL, anastomotic leakage; C, control group; F, Female; I, intervention group; M, Male; LAR, low anterior resection; PSM, propensity-score matched study; N, not available; RCT, randomized controlled trial; USA, the United States of America*.

### Quality Assessment

**Figure 2:**
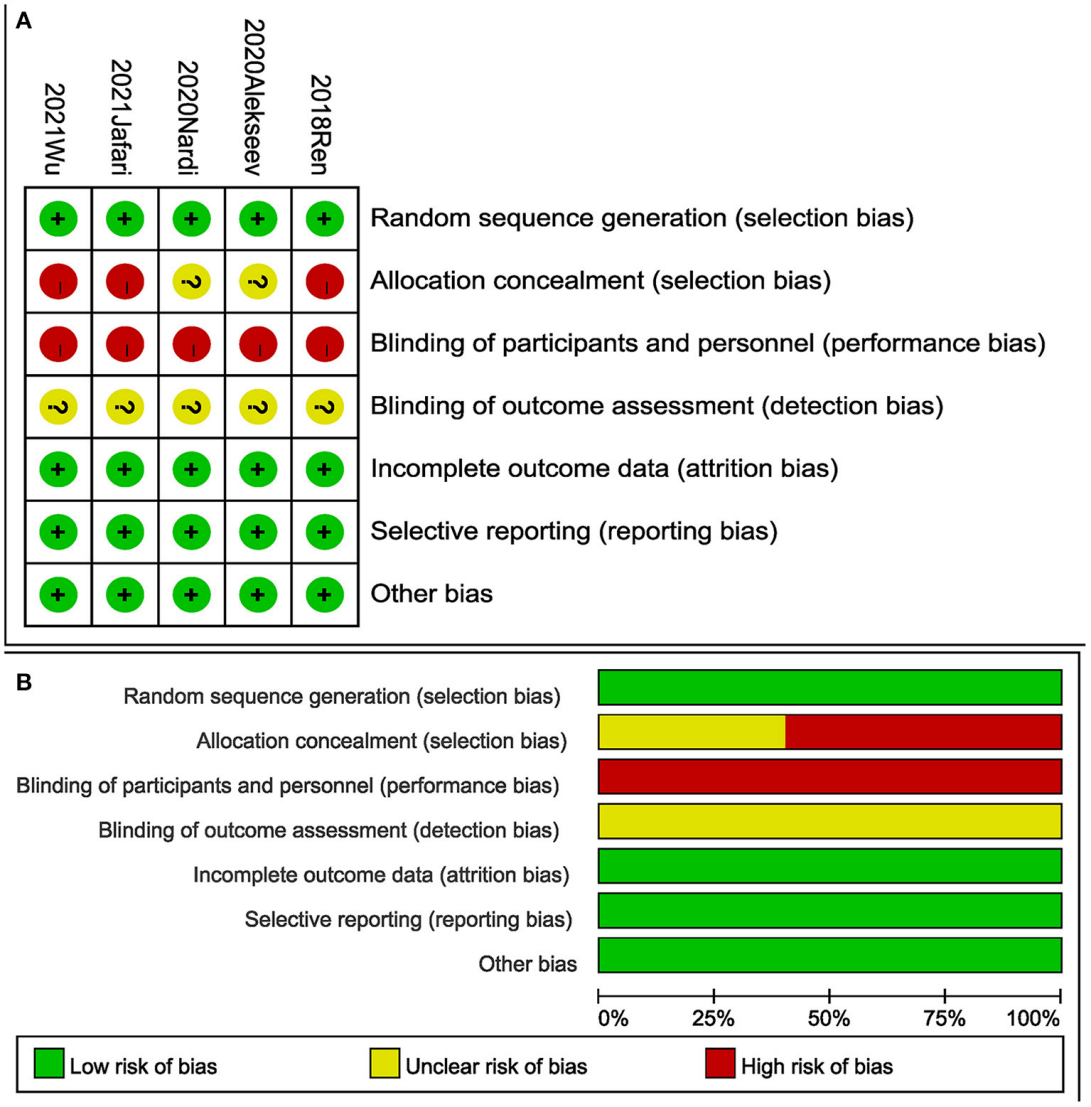
Risk of bias for each included study. **(A)** Risk of bias summary. **(B)** Risk of bias graph.

Fifteen trials were evaluated to be of good quality based on the NOS (Table 2) with scores of 6 and more. The risk of bias of RCTs is shown in Figure 2. The 5 RCTs were assessed to be of low risk.

**Table 2:** Outcome of assessment of the quality of non-randomized studies using the Newcastle-Ottawa scale.

**Reference**	**Selection**				**Comparability**	**Outcome**			**Total score**
	**Representativeness of the exposed cohort**	**Selection of non-exposed cohort**	**Ascertainment of exposure**	**Outcome not presented at the start**		**Assessment of outcome**	**Follow-up long enough**	**Adequacy of follow up**	
Kudszus et al. (29)	*	-	*	*	**	*	-	-	6/9
Kin et al. (30)	*	-	*	*	**	*	*	*	8/9
Boni et al. (31)	*	-	*	*	**	*	-	-	6/9
Mizrahi et al. (32)	*	-	*	*	**	*	*	*	8/9
Wada, et al. (34)	*	-	*	*	**	*	-	*	7/9
Ishii et al. (35)	*	*	*	*	**	*	-	*	8/9
Kojima et al. (36)	*	-	*	*	**	*	-	*	7/9
Spinelli et al. (37)	*	-	*	*	**	*	*	*	8/9
Watanabe et al. (45)	*	*	*	*	**	*	-	*	8/9
Losurdo et al. (39)	*	-	*	*	**	*	-	*	7/9
Foo et al. (26)	*	-	*	*	**	*	*	*	8/9
Hasegawa et al. (38)	*	*	*	*	**	*	-	*	8/9
Wojcik et al. (41)	*	*	*	*	**	*	-	*	8/9
Watanabe et al. (42)	*	*	*	*	**	*	-	*	8/9
Yanagita et al. (44)	*	-	*	*	**	*	-	*	7/9

A single asterisk (^*^) indicates 1 score, ** indicates 2 score, and dash (-) indicates 0 score.

### Meta-Analysis

#### AL Rate

**Figure 3:**
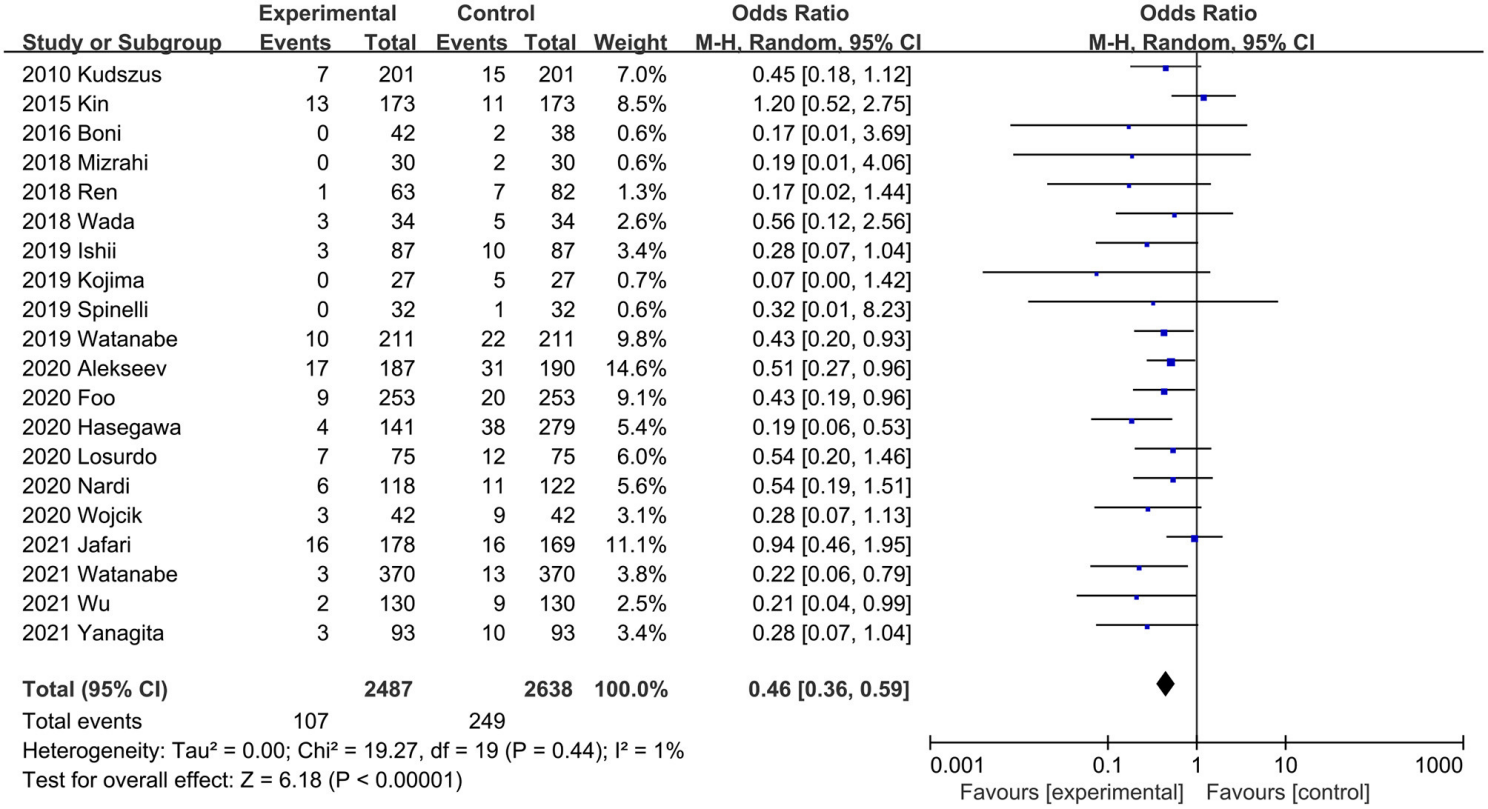
Effect of indocyanine green on anastomotic leakage rate.

AL rate was reported in all 20 studies (20, 21, 26, 29–45). Compared with the control group, the incidence of AL was significantly reduced in the ICG group (OR, 0.46; 95% CI, 0.36, 0.59; *P* < 0.00001). No significant heterogeneity was observed (*P* = 0.44; *I*^2^ = 1%) (Figure 3). The results of subgroup analysis showed that ICG could effectively reduce the incidence of AL in both RCTs (20, 21, 33, 40, 43) (OR, 0.55; 95% CI, 0.34, 0.88; *P* = 0.01; *I*^2^ = 17%) (Table 3) and PSM studies (26, 29–32, 34–39, 41, 42, 44, 45) (OR, 0.41; 95% CI, 0.30, 0.56; *P* < 0.00001; *I*^2^ = 0%) (Table 3). When subgroups were performed according to surgical methods, ICG could effectively reduce the incidence of AL regardless of colorectal surgery (20, 26, 29, 30, 33, 35, 36, 38–44) (OR, 0.45; 95% CI, 0.34, 0.61; *P* < 0.00001; *I*^2^ = 0%) (Table 3) or low anterior resection (21, 31, 32, 34, 37, 38, 45) (OR, 0.45; 95% CI, 0.26, 0.78; *P* = 0.004; *I*^2^ = 21%) (Table 3).

**Table 3:** Summary of results from all subgroup analyses.

**Outcome**	**Subgrouped by**	**The number of studies**	**Effect size**	**95%CI**	***I^**2**^* (%)**	**P for between subgroup heterogeneity**
**AL**	Surgery type	-	-	-	-	0.27
	Colorectal resection	13	0.45	0.34, 0.61	0	-
	Low anterior resection	7	0.45	0.26, 0.78	21	-
	Study type	-	-	-	-	0.32
	PSM	15	0.41	0.30, 0.56	0	-
	RCT	5	0.55	0.34, 0.88	17	-
**SAL**	Surgery type	-	-	-	-	0.66
	Colorectal resection	6	0.51	0.32, 0.82	4	-
	Low anterior resection	4	0.43	0.22, 0.82	0	-
	Study type	-	-	-	-	0.08
	PSM	8	0.39	0.25, 0.61	0	-
	RCT	2	0.81	0.41, 1.61	0	-
**Postoperative morbidity**	Surgery type	-	-	-	-	0.16
	Colorectal resection	5	0.77	0.56, 1.05	10	-
	Low anterior resection	6	1.31	0.66, 2.61	78	-
	Study type	-	-	-	-	0.23
	PSM	8	1.10	0.62, 1.95	73	-
	RCT	3	0.74	0.53, 1.02	0	-
**Postoperative ileus**	Surgery type	-	-	-	-	0.46
	Colorectal resection	3	1.82	0.65, 5.11	0	-
	Low anterior resection	4	1.00	0.29, 3.44	51	-
	Study type	-	-	-	-	0.49
	PSM	4	1.93	0.57, 6.50	7	-
	RCT	3	1.06	0.32, 3.54	58	-
**Wound infection**	Surgery type	-	-	-	-	0.19
	Colorectal resection	4	0.60	0.32, 1.15	0	-
	Low anterior resection	4	1.38	0.49, 3.89	0	-
	Study type	-	-	-	-	0.52
	PSM	6	0.84	0.45, 1.57	0	-
	RCT	2	0.52	0.15, 1.89	16	-
**Anastomotic bleeding**	Surgery type	-	-	-	-	0.36
	Colorectal resection	2	0.59	0.02, 19.75	73	-
	Low anterior resection	2	3.72	0.60, 22.96	0	-
**Reoperation**	Surgery type	-	-	-	-	0.30
	Colorectal resection	5	0.82	0.42, 1.58	47	-
	Low anterior resection	3	0.35	0.08, 1.51	14	-
	Study type	-	-	-	-	0.10
	PSM	6	0.52	0.26, 1.07	32	-
	RCT	2	1.29	0.59, 2.84	0	-
**Conversion rates**	Surgery type	-	-	-	-	0.98
	Colorectal resection	3	1.35	0.16, 11.78	21	-
	Low anterior resection	3	1.32	0.60, 2.91	0	-
**Mortality**	Surgery type	-	-	-	-	0.48
	Colorectal resection	4	0.36	0.07, 1.77	0	-
	Low anterior resection	2	0.98	0.10, 9.54	0	-
	Study type	-	-	-	-	0.54
	PSM	3	0.75	0.12, 4.84	0	-
	RCT	3	0.33	0.05, 2.10	0	-
**Operative time**	Surgery type	-	-	-	-	0.03
	Colorectal resection	7	1.78	−2.48, 6.03	23	-
	Low anterior resection	5	−24.18	−47.85, −0.52	91	-
	Study type	-	-	-	-	0.09
	PSM	9	−14.45	−31.52, 2.62	91	-
	RCT	3	0.94	−4.06, 5.95	23	-
**Blood loss**	Surgery type	-	-	-	-	0.68
	Colorectal resection	4	−3.87	−7.54, −0.21	54	-
	Low anterior resection	2	−18.60	−89.49, 52.29	86	-
	Study type	-	-	-	-	0.63
	PSM	4	−10.20	−43.38, 22.99	90	-
	RCT	2	−1.97	−4.81, 0.87	0	-
**Postoperative hospital stay**	Surgery type	-	-	-	-	0.49
	Colorectal resection	7	−1.10	−2.05, −0.16	86	-
	Low anterior resection	2	−1.78	−3.46, −0.10	0	-
	Study type	-	-	-	-	0.32
	PSM	6	−1.67	−2.90, −0.43	65	-
	RCT	3	−0.61	−2.28, 1.05	87	-

*AL, anastomotic leakage; LAR, low anterior resection; PSM, propensity-score matched study; RCT, randomized controlled trial; SAL, Symptomatic anastomotic leakage*.

**Figure 4:**
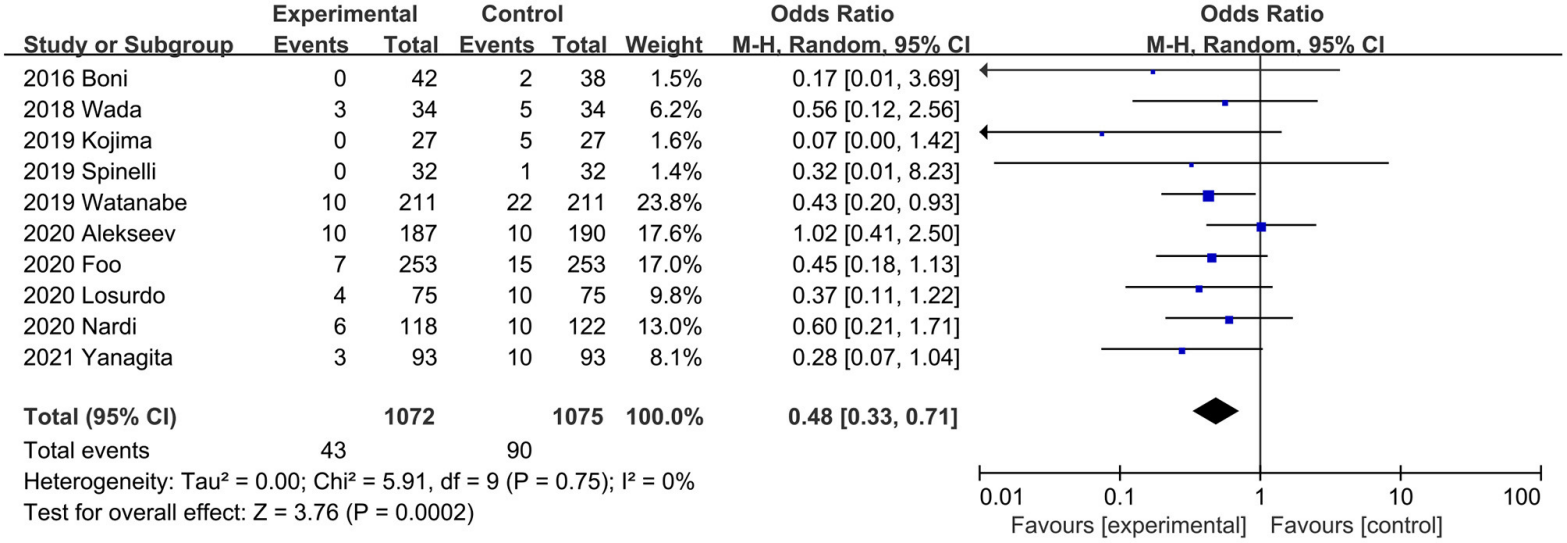
Effect of indocyanine green on symptomatic anastomotic leakage rate.

Ten studies (20, 26, 31, 34, 36, 37, 39, 40, 44, 45) described the incidence of SAL. Data from RCTs and PSM studies showed that ICG was associated with a lower risk of SAL, with low heterogeneity between studies (OR, 0.48; 95% CI, 0.33, 0.71; *P* = 0.0002; *I*^2^ = 0%) (Figure 4).

#### Postoperative Complications

**Figure 5:**
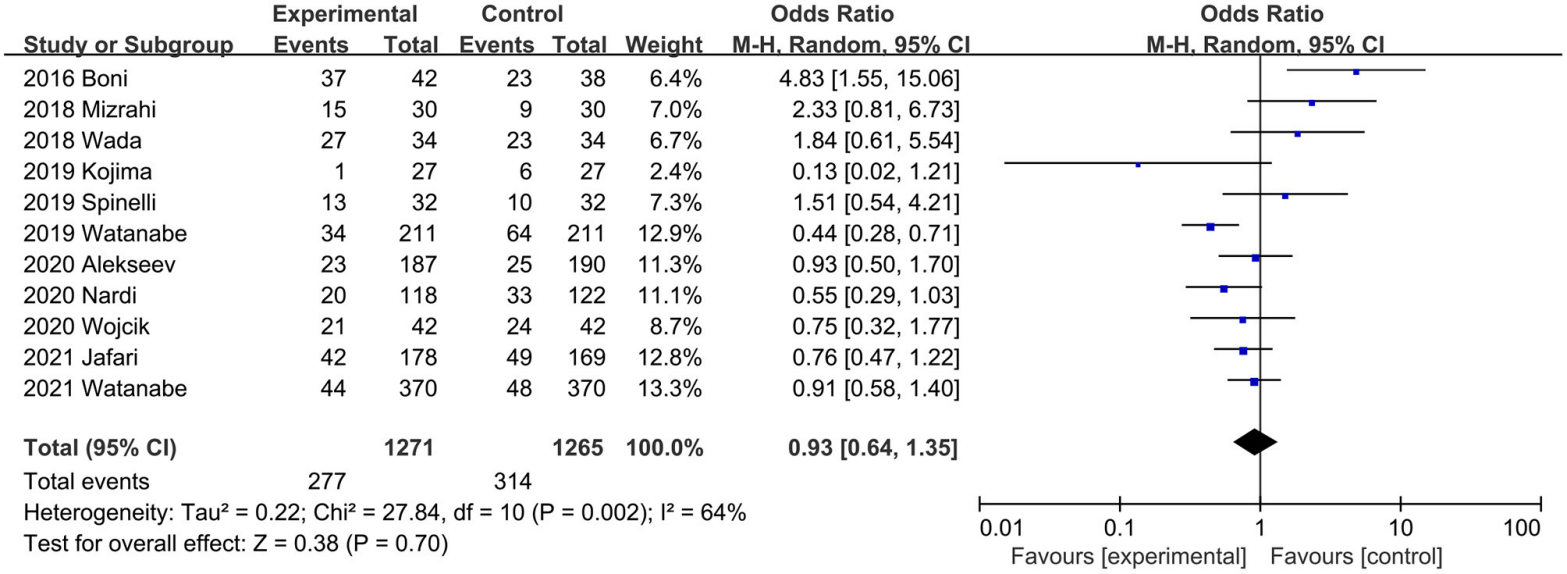
Effect of indocyanine green on total postoperative complications rate.

Postoperative complications were described in 11 studies (20, 21, 31, 32, 34, 36, 37, 40–42, 45). The total effect size indicated that intraoperative ICG fluorescence angiography did not reduce the incidence of total complications, with significant heterogeneity between studies (OR, 0.93; 95% CI, 0.64, 1.35; *P* = 0.70; *I*^2^ = 64%) (Figure 5). When subgroup analysis was performed by study type, the combined effect size of both RCTs (20, 21, 40) (OR, 0.74; 95% CI, 0.53, 1.02; *P* = 0.06) (Table 3) and PSM studies (31, 32, 34, 36, 37, 41, 42, 45) (OR, 1.10; 95% CI, 0.62, 1.95; *P* = 0.75) (Table 3) showed that ICG did not increase the incidence of total postoperative complications, and heterogeneity in the RCTs subgroup was significantly reduced (*P* = 0.50; *I*^2^ = 0%) (Table 3).

#### Postoperative Ileus

**Figure 6:**
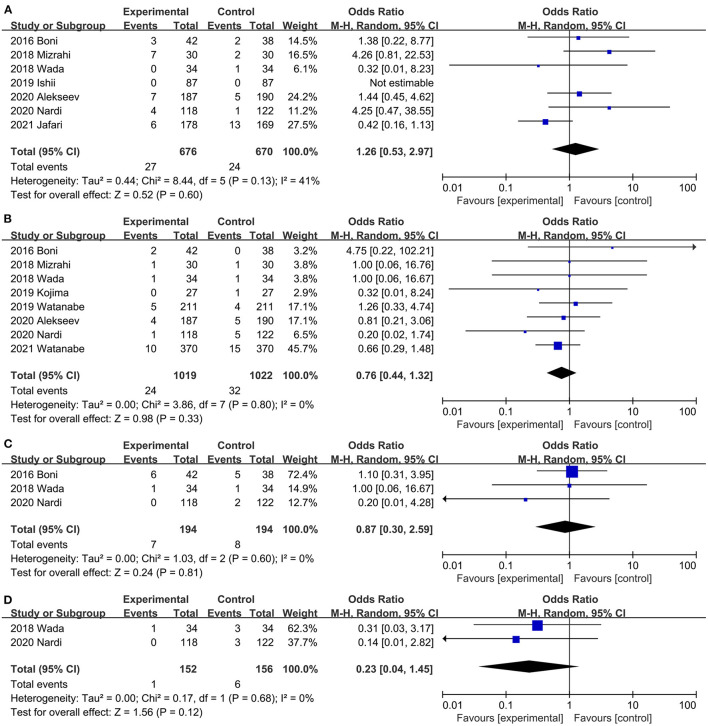
Effect of indocyanine green on **(A)** Postoperative ileus rate. **(B)** Wound infection rate **(C)** Anastomotic stricture rate. **(D)** Pulmonary infection rate.

Evidence from a combination of 7 studies (20, 21, 31, 32, 34, 35, 40) suggests that ICG does not reduce the incidence of postoperative ileus, and no significant heterogeneity was observed between studies (OR, 1.26; 95% CI, 0.53, 2.97; *P* = 0.60; *I*^2^ = 41%) (Figure 6A). When subgroup analysis was based on study type, both RCTs (20, 21, 40) (OR, 1.06; 95% CI, 0.32, 3.54; *P* = 0.93; *I*^2^ = 58%) (Table 3) and PSM studies (31, 32, 34, 35) (OR, 1.93; 95% CI, 0.57, 6.50; *P* = 0.29; *I*^2^ = 7%) (Table 3) showed that ICG did not reduce the incidence of postoperative intestinal obstruction. There was no significant heterogeneity between subgroups (*P* = 0.49; *I*^2^ = 0%) (Table 3).

#### Wound Infection

Postoperative wound infection was reported in 8 studies (20, 31, 32, 34, 36, 40, 42, 45) (2 RCTs, 6 PSM studies), ICG did not reduce the risk of postoperative wound infection, and there was no significant heterogeneity between studies (OR, 0.76; 95% CI, 0.44, 1.32; *P* = 0.33; *I*^2^ = 0%) (Figure 6B). Both RCTs (20, 40) (OR, 0.52; 95% CI, 0.15, 1.89; *P* = 0.32; *I*^2^ = 16%) (Table 3) and PSM studies (31, 32, 34, 36, 42, 45) (OR, 0.84; 95% CI, 0.45, 1.57; *P* = 0.58; *I*^2^ = 0%) (Table 3) showed that ICG does not reduce the incidence of postoperative wound infection. Subgroup analysis showed that ICG did not reduce the incidence of postoperative wound infection during colorectal surgery (20, 36, 40, 42) (OR, 0.60; 95% CI, 0.32, 1.15; *P* = 0.13; *I*^2^ = 0%) (Table 3) or low anterior resection (31, 32, 34, 45) (OR, 1.38; 95% CI, 0.49, 3.89; *P* = 0.55; *I*^2^ = 0%) (Table 3).

#### Urinary Tract Infection

Three studies (20, 31, 34) reported the urinary tract infections of both groups, and the difference between the ICG group and control group was not statistically significant (OR, 0.87; 95% CI, 0.30, 2.59; *P* = 0.81) (Figure 6C). No significant heterogeneity was observed (*P* = 0.60; *I*^2^ = 0%) (Figure 6C).

#### Pulmonary Infection

Pulmonary infection was reported in two studies (20, 34). Results of the meta-analysis showed that ICG did not reduce the incidence of pulmonary infection (OR, 0.23; 95% CI, 0.04, 1.45; *P* = 0.12) (Figure 6D), and there was no significant heterogeneity between studies (*P* = 0.68; *I*^2^ = 0%) (Figure 6D).

#### Urinary Retention

**Figure 7:**
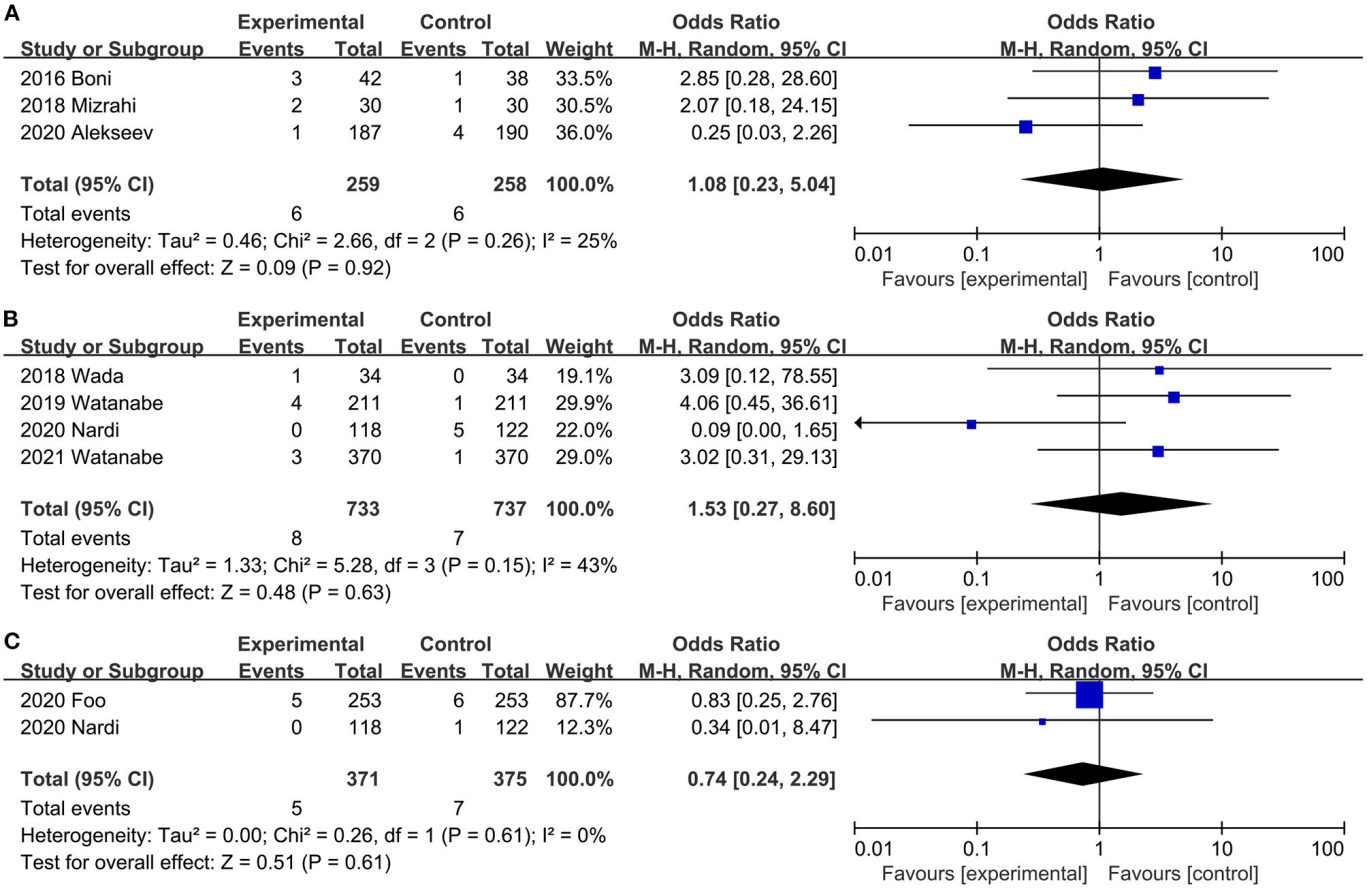
Effect of indocyanine green on **(A)** Urinary retention rate. **(B)** Anastomotic bleeding rate **(C)** Urinary tract infection rate.

A combined dataset of 517 participants from three studies (31, 32, 40) showed that ICG did not reduce the risk of postoperative urinary retention (OR, 1.08; 95% CI, 0.23, 5.04; *P* = 0.92) (Figure 7A). No significant heterogeneity was observed (*P* = 0.26; *I*^2^ = 25%) (Figure 7A).

#### Anastomotic Bleeding

Four studies (20, 34, 42, 45) reported the rate of anastomotic bleeding. Intraoperative ICG fluorescence angiography did not reduce (OR, 1.53; 95% CI, 0.27, 8.60; *P* = 0.63) (Figure 7B) the incidence of anastomotic bleeding, and there was no significant heterogeneity (*P* = 0.15; *I*^2^ = 43%) (Figure 7B) between studies.

#### Anastomotic Stricture

Two trials (20, 26) reported the Incidence of anastomotic stricture. There was no significant difference in the Incidence of anastomotic stricture between the ICG and the control groups (OR, 0.74; 95% CI, 0.24, 2.29; *P* = 0.61) (Figure 7C). No significant heterogeneity (*P* = 0.61; *I*^2^ = 0%) (Figure 7C) was observed.

#### Reoperation Rates

**Figure 8:**
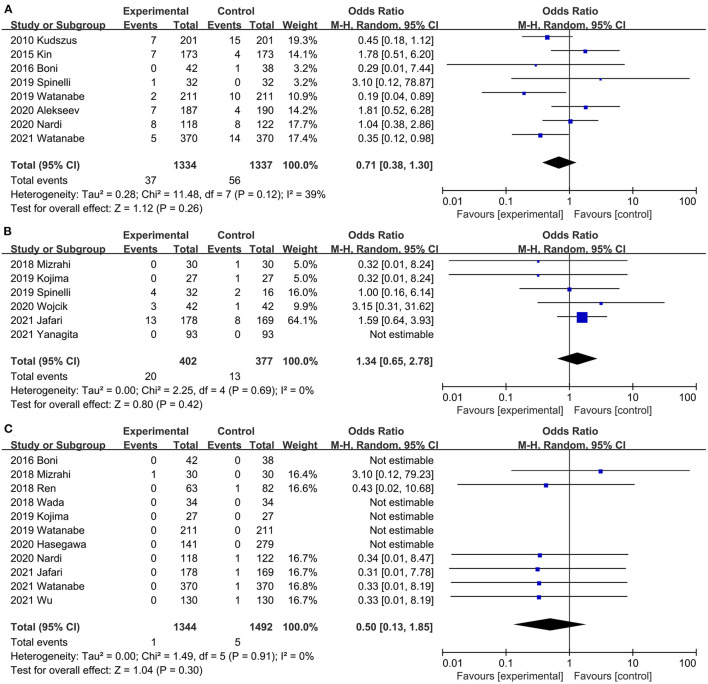
Effect of indocyanine green on **(A)** Reoperation rate rate. **(B)** Conversion rate **(C)** Mortality.

Eight studies (20, 29–31, 37, 40, 42, 45) assessed the effect of ICG on postoperative reoperation rates. The combined effect size showed a lower reoperation rate in the ICG group than in the control group, but the difference was not statistically significant (OR, 0.71; 95% CI, 0.38, 1.30; *P* = 0.26; *I*^2^ = 39%) (Figure 8A). Subgroup analysis showed that ICG did not reduce reoperation rate in both RCTs (20, 40) (OR, 1.29; 95% CI, 0.59, 2.84; *P* = 0.52; *I*^2^ = 0%) (Table 3) and PSM studies (29–31, 37, 42, 45) (OR, 0.52; 95% CI, 0.26, 1.07; *P* = 0.08; *I*^2^ = 32%) (Table 3).

#### Conversion Rates

Six studies (21, 32, 36, 37, 41, 44) mentioned conversion rates. ICG did not increase conversion rates during surgery compared with the control group (OR, 1.34; 95% CI, 0.65, 2.78; *P* = 0.42) (Figure 8B), with no significant heterogeneity between studies (*P* = 0.69; *I*^2^ = 0%) (Figure 8B).

#### Mortality

Postoperative mortality was reported in 11 studies (20, 21, 31–34, 36, 38, 42, 43, 45). There was no significant difference in perioperative mortality (OR, 0.50; 95% CI, 0.13, 1.85; *P* = 0.30) (Figure 8C) between the ICG group and the control group, and no significant heterogeneity (*P* = 0.91; *I*^2^ = 0%) (Figure 8C) was observed between studies.

#### Operative Time

**Figure 9:**
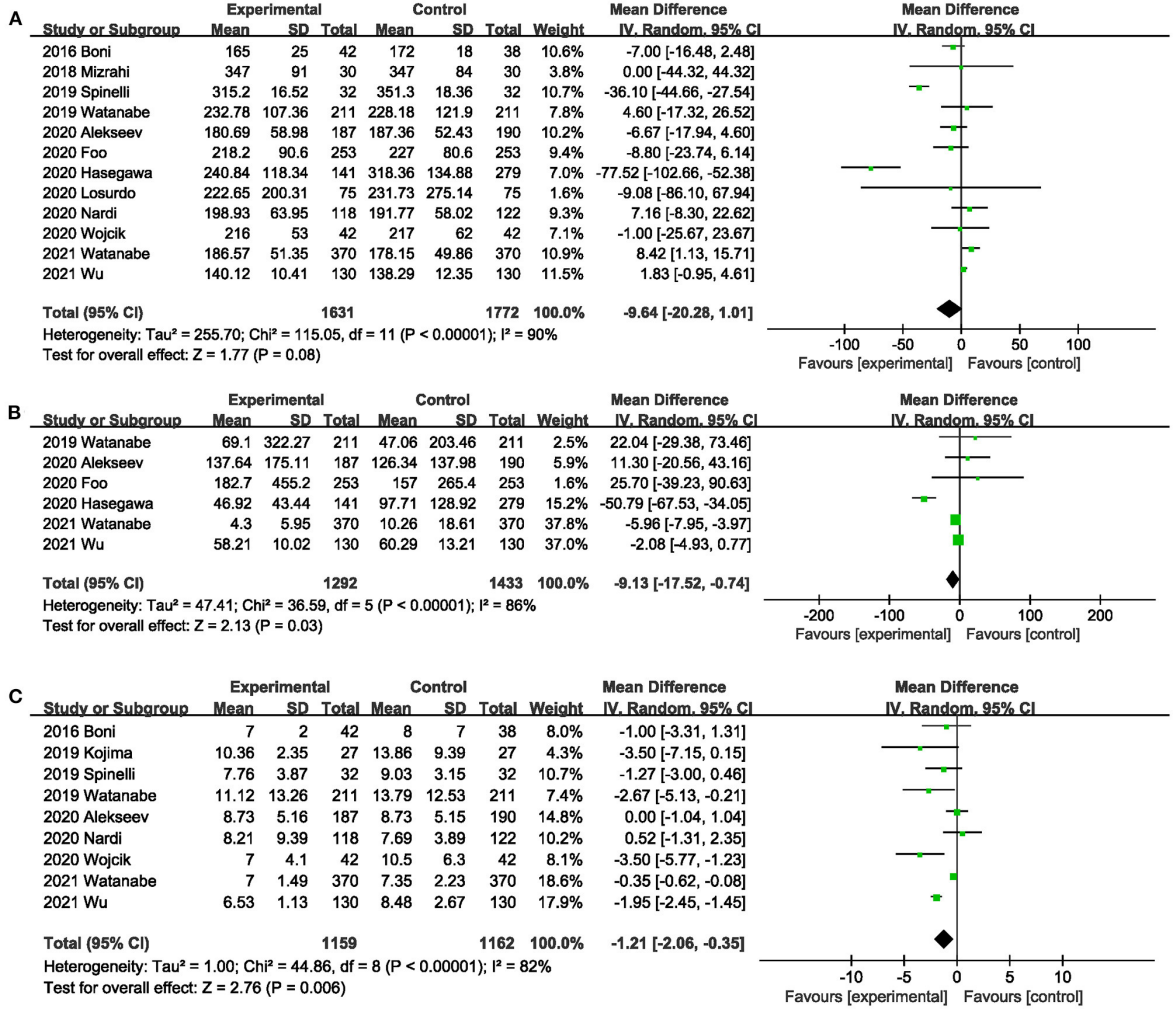
Effect of indocyanine green on **(A)** Operative time. **(B)** Blood loss during surgery. **(C)** Length of postoperative hospital stay.

Twelve studies (20, 26, 31, 32, 37–43, 45) compared the operative time between the ICG group and the control group. The total effect size showed that intraoperative ICG fluorescein angiography did not increase the operative time (MD,−9.64; 95% CI,−20.28, 1.01; *P* = 0.08) (Figure 9A), and significant heterogeneity was observed between studies (*P* < 0.00001; *I*^2^ = 90%) (Figure 9A). Subgroup analysis based on study type found that heterogeneity significantly decreased between RCTs (20, 40, 43) (*P* = 0.27; *I*^2^ = 23%) (Table 3), and heterogeneity was significant (*P* = 0.09; *I*^2^ = 65.2%) (Table 3) between subgroups.

#### Blood Loss

Six studies (26, 38, 40, 42, 43, 45) reported the blood loss during surgery. ICG can effectively reduce the blood loss during surgery (MD,−9.13; 95% CI,−17.52,−0.74; *P* = 0.03) (Figure 9B). Significant heterogeneity (*P* < 0.00001; *I*^2^ = 81%) (Figure 9B) was observed between these studies. When subgroup analysis was performed by type of surgery, intraoperative ICG fluorescein angiography did not reduce the amount of blood loss during low anterior resection (38, 45) (MD,−18.60; 95% CI,−89.49, 52.29; *P* = 0.61; *I*^2^ = 86%) (Table 3), but it did reduce the blood loss during colorectal surgery (26, 40, 42, 43) (MD,−3.87; 95% CI,−7.54,−0.21; *P* = 0.04; *I*^2^ = 54%) (Table 3).

#### Length of Postoperative Hospital Stay

Nine studies (20, 31, 36, 37, 40–43, 45) reported length of postoperative hospital stay. Meta-analysis showed that intraoperative ICG fluorescence angiography could effectively shorten postoperative hospital stay (MD,−1.21; 95% CI,−2.06,−0.35; *P* = 0.06) (Figure 9C), with significant heterogeneity among 9 studies (*P* < 0.00001; *I*^2^ = 82%) (Figure 9C). When subgroup analysis was performed based on study type, benefits of ICG for shorter length of hospital stay were observed only in the PSM studies (31, 36, 37, 41, 42, 45) (MD,−1.67; 95% CI,−2.90,−0.43; *P* = 0.008; *I*^2^ = 65%) (Table 3).

### Sensitivity Analysis

The results of the sensitivity analysis showed that no single trial could affect the total effect size of AL rate, SAL rate, postoperative complications, postoperative ileus, wound infection, urinary tract infection, pulmonary infection, urinary retention, anastomotic bleeding, anastomotic stricture, conversion rates, reoperation rate, length of postoperative hospital stay, mortality and operative time. The study of Watanabe et al. (42) (MD,−4.90; 95% CI,−33.76, 23.97; *P* = 0.74; *I*^2^ = 88%) and the study of Zhang et al. (8) (MD,−6.55; 95% CI,−33.83, 20.72; *P* = 0.64; *I*^2^ = 87%) significantly affected the effect size of blood loss during surgery.

### Publication Bias

**Figure 10:**
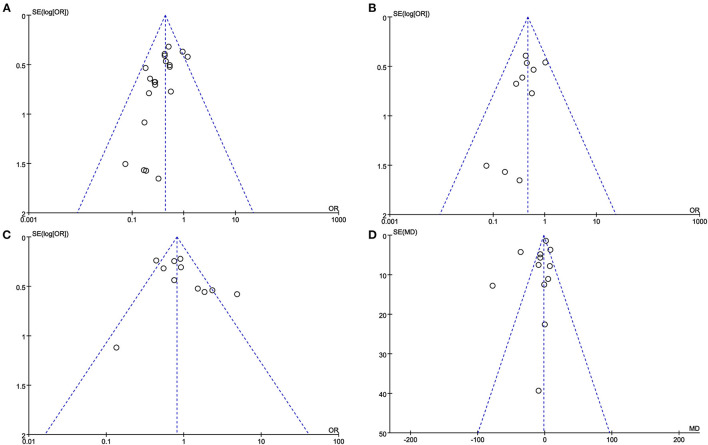
The funnel plot of **(A)** AL rate. **(B)** SAL rate. **(C)** Postoperative complications. **(D)** Blood loss during surgery.

The funnel plot of AL rate, SAL rate, postoperative complications and blood loss during surgery reveals a roughly symmetrical distribution of studies (Figure 10).

## Discussion

AL has increased the medical burden of patients and caused destructive results (4), so it is necessary to find effective strategies to reduce the risk of AL after colorectal surgery. In 2010, Kudszus et al. (29) first reported that ICG reduced the occurrence of AL after colorectal surgery by 4%. Skrovina et al. (18) also confirmed that ICG fluorescence angiography may be a potential strategy for preventing AL. Impellizzeri et al. (16) found that ICG fuorescence angiography is associated with a lower risk of AL after colorectal cancer surgery. The evidence from the above clinical studies well supports our conclusions. However, in Dinallo et al. study (46), the incidence of AL after colorectal surgery was 1.3% in both the ICG group and the non-ICG group. The low incidence of AL in the study may mask the true effect of ICG. In addition, almost all recent meta-analyses (4, 8, 22, 23) on this topic showed that intraoperative ICG fluorescence angiography could reduce the incidence of postoperative AL.

Our meta-analysis showed that ICG can effectively reduce the AL rate, SAL rate, blood loss, and hospital stays, without prolonging the operation time or increasing postoperative complications in colorectal surgery. The results of subgroup analysis indicated that both evidence from RCTs and PSM studies evidence indicated that ICG fluorescence angiography was an effective strategy for reducing postoperative AL. Although the incidence of asymptomatic AL is as high as 14%, the use of contrast agents to detect asymptomatic AL in post-colorectal surgery patients is not a routine strategy in clinical practice (4). Asymptomatic AL has little damage to the prognosis of patients, and almost all asymptomatic AL do not need intervention. In contrast, SAL was associated with poor short-and long-term outcomes of colorectal surgery (4). Therefore, we evaluated the preventive effect of ICG on SAL separately. We found that ICG use was associated with a reduced incidence of SAL. Previous studies have shown that the incidence of AL is related to the position of the anastomotic, and the lower the position, the higher the risk of AL (23, 47). Therefore, the trial of low anterior resection was used as a subgroup in this study, and the results of subgroup analysis showed that ICG could effectively reduce the incidence of AL in this high-risk population. Similarly, a retrospective study by Jafari et al. (15) found that the risk of AL in robot-assisted rectal surgery was reduced to 6% in the ICG group, compared with 18% in the control group. In a meta-analysis that included 27 studies, Emile et al. (48) found that ICG was associated with a significant reduction in the incidence of AL, whether in a subgroup analysis based on RCTs or in a subgroup analysis based on studies that included rectal cancer only. AL could lead to prolonged hospital stay (49). The results of this study showed that ICG could shorten the hospital stay of patients, which may be related to the reduction of the occurrence of AL. Grade C AL often requires surgical intervention, and the study of Liu et al. (22) showed that ICG could reduce the reoperation rate. However, no benefit of ICG in reducing reoperation rates was observed in this study. This may be related to the fact that few studies reported relevant outcome measures, with only eight of the included studies describing reoperation rates. In addition, our results suggest that ICG does not reduce postoperative mortality, which may be related to the low incidence of perioperative mortality and the small sample size of some of the included studies. Future prospective studies with a larger sample size should be conducted to investigate whether ICG fluorescein reduces the risk of perioperative mortality in colorectal surgery.

ICG is a safe dye, and its adverse reactions are rarely reported (50, 51). In a study of 1,226 participants, adverse events were observed in only eight subjects after intravenous ICG administration of 1 to 5 mg/kg, with only one severe adverse event and no deaths reported (52). Su et al. (50) found that no adverse reactions or allergic reactions associated with ICG were observed in colon cancer patients injected with 15 mg ICG. The doses used in the trials included in this study ranged from 0.1 to 0.5 mg/kg, and no adverse reactions were reported. In colorectal cancer surgery, Manen et al. (53) recommended intravenous injection of low-dose (2.5 mg) ICG to prevent AL, because 2.5 mg ICG can clearly observe the situation of colorectal anastomosis. Three studies (30, 34, 37) included in this study using 5mg of ICG showed that 5mg of ICG was effective in reducing the incidence of AL associated with perfusion. Although low-dose ICG may be an effective strategy to reduce AL, it is not clear whether low-dose ICG and high-dose ICG are equally effective in preventing AL. Our study showed that intraoperative ICG fluorescence angiography did not increase the incidence of total postoperative complications. Compared with the control group, ICG did not increase the risk of postoperative intestinal obstruction, wound infection, pulmonary infection, urinary retention, anastomotic bleeding, and anastomotic stenosis. A recent meta-analysis by Zhang et al. (8) showed that ICG fluorography did not increase wound infection, pneumonia, urinary retention, mortality, or postoperative bleeding. In addition, the results of this study showed that intraoperative ICG angiography did not prolong the operative time, but rather reduced intraoperative blood loss compared with the control group. This may be due to the increased frequency with which ICG fluorescein angiography was used, resulting in surgeons becoming more proficient with the system (23). A meta-analysis of 23 studies also showed that ICG did not increase intraoperative blood loss or operative time (9).

This study has several strengths. First, in order to reduce potential bias, this study conducted a comprehensive literature search of several electronic databases (Embase, China National Knowledge Infrastructure, Web of Science, Scopus, PubMed, Cochrane Library, and VIP databases) without any language or time restrictions. Second, several recent important studies were included, which made our evidence more convincing. Third, different from previous meta-analyses, we only included PSM studies and RCTs, which made the experimental group and the control group more comparable and strengthened the reliability of our conclusions. Finally, advanced statistical methods (sensitivity analysis and subgroup analysis) were used to further confirm the robustness of our results.

There are several limitations in our meta-analysis. First, there was significant heterogeneity in some outcome measures of this study. This may be related to inconsistent follow-up times (from 30 to 90 days) and inconsistent definitions of AL used in the included studies. Moreover, five studies included patients with both malignant and benign colorectal disease. Inconsistent disease types may be one of the sources of heterogeneity. Second, a total of nine fluorescence imaging systems were used. It is not clear whether the effects of different fluorescence imaging systems are consistent, which may need to be clarified in future studies. In the included studies, there were also differences in the dose of ICG injected intravenously. The influence of different doses on the study needs to be further explored, and finding the optimal dose may be the focus of future studies. These may also be sources of heterogeneity. Third, although this study showed that ICG may have potential benefits in reducing the incidence of AL after colorectal surgery, the fluorescence intensity in all the studies included in this meta-analysis was based on the subjective judgment of surgeons, lacking objective evaluation indicators (54). In addition, even if ICG fluorescence is displayed in the colorectal, intestinal ischemia may occur if blood flow is not meeting physiological demands (55). Therefore, the use of software to quantify the fluorescence parameters and find reliable parameters for predicting AL (54) may further confirm the benefits of ICG on AL in colorectal surgery. Cahill et al. (56) combined ICG fluorescence angiography and artificial intelligence to identify tumors by recognizing different perfusion modes. This technology can also be developed into real-time monitoring of anastomotic blood perfusion (57), so as to identify ischemic anastomotic sites. Finally, some of the outcome indicators (reoperation rate, conversion rate, postoperative ileus rate, wound infection rate, urinary tract infection rate, pulmonary infection rate, urinary retention rate, anastomotic bleeding rate and anastomotic stricture rate) in the included studies were based on evidence from a small number of studies, so it is not possible to determine whether ICG will bring more benefits, and more high-quality studies are needed to explore the impact of ICG on these outcomes.

## Conclusion

In conclusion, this meta-analysis demonstrated the value of ICG in patients undergoing colorectal surgery, as evidenced by the reduced AL rate, SAL rate, and blood loss. Further, hospital stays were shorter. ICG may be a potential strategy to prevent AL in colorectal surgery, and more high-quality large sample size RCTs are necessary to confirm the benefits of ICG in colorectal surgery.

## Data Availability Statement

The original contributions presented in the study are included in the article/Supplementary Material, further inquiries can be directed to the corresponding author.

## Author Contributions

ZW, GT, JT, and DD: conceptualization and had primary responsibility for final content. JT, GT, and DD: data collection, analyses, and writing—original draft preparation. ZW, GT, and DD: writing—review and editing. All authors contributed to the article and approved the submitted version.

## Funding

This study was funded by Chongqing Joint Medical Scientific Research Project of Science and Health (No. 2018ZDXM007) and Chongqing Key Diseases Research and Application Demonstration Program (No. 2019ZX003).

## Conflict of Interest

The authors declare that the research was conducted in the absence of any commercial or financial relationships that could be construed as a potential conflict of interest.

## Publisher's Note

All claims expressed in this article are solely those of the authors and do not necessarily represent those of their affiliated organizations, or those of the publisher, the editors and the reviewers. Any product that may be evaluated in this article, or claim that may be made by its manufacturer, is not guaranteed or endorsed by the publisher.
